# Treatment of acute acquired concomitant esotropia

**DOI:** 10.1186/s12886-020-01787-1

**Published:** 2021-01-06

**Authors:** Minghua Shi, Yuanxiang Zhou, Aijiao Qin, Jing Cheng, Hongxing Ren

**Affiliations:** 1 Department of Strabismus and Pediatric Opthalmology, Wuhan Aier Eye Hospital(Hanyang), Wuhan, 430020 China; 2 Department of Strabismus and Pediatric Opthalmology, Yueyang Aier Eye Hospital, Yueyang, 414000 China

**Keywords:** Acute acquired concomitant esotropia, Botulinum toxin, Extraocular muscle surgery, Squint

## Abstract

**Background:**

The treatment efficacy of botulinum toxin bilateral medial rectus injections for acute acquired concomitant esotropia (AACE) in adult is not clear. We characterize the effects of botulinum toxin injection in the treatment of AACE, especially in patients over 14 years old, and compared it with surgical treatment.

**Methods:**

In this prospective, nonrandomized, controlled clinical study, patients with AACE in our hospital from March 2017 to March 2020 elected to receive bilateral medial rectus injections of botulinum toxin or to undergo extraocular muscle surgery. Ocular position and stereopsis were evaluated before and after treatment.

**Results:**

A total of 60 patients were treated: 40 patients in the botulinum toxin group, and 20 patients in the surgery group. The botulinum toxin group included 31 cases ≥ 14 years of age and 9 cases < 14 years of age. After 1–3 botulinum injections, the cumulative initial success rate was 95% (38/40), and the recurrence rate was 22.5% (9/40). Nine children < 14 years of age were treated successfully, without recurrence. In the surgery group, the initial success rate after surgery was 75% (17/20), and the recurrence rate was 20% (4/20). There was no significant difference between groups in the rate of success rate or the rate of recurrence (*P* > 0.05).

**Conclusion:**

The injection of botulinum toxin has a good effect on AACE in adults and children. The outcomes achieved with injected botulinum toxin are similar to those achieved with surgery.

**Trial registration:**

ChiCTR, ChiCTR2000032544. Registered May 2, 2020, Retrospectively registered.

## Background

The incidence rate of acute acquired concomitant esotropia has increased dramatically, especially in Asian countries [1–5]. There are various treatments for AACE, includes extraocular muscle surgery, botulinum toxin injection, prisms, and divergence training. However, no standard protocol for treatment has been established. Extraocular muscle surgery remains the main treatment method. However, because of the “eating-prism phenomenon”, it is difficult to accurately correct the target deviation [6–10]. Many authors have published satisfactory results in the treatment of various types of strabismus in children, including AACE, with botulinum toxin [1, 2, 9, 11–19]. However, the effects of this approach to treatment remain to be evaluated in an adult patient population. Recently, some reports on treating AACE with botulinum toxin included some adult patients, but because the number of cases is small, the authors did not discuss the effects of this approach to treatment on adults in particular [1, 2]. The efficacy of this approach needs to be further investigated. We investigated the efficacy of botulinum toxin injection, compared with the conventional surgical approach, in the treatment of AACE, especially in patients ≥ 14 years of age.

## Methods

From March 2017 to March 2020, all AACE patients admitted to our hospital were included in this study. The diagnostic criteria were: sudden-onset esotropia with diplopia, same esotropia angle in each direction, and normal eye movements in each direction. Patients with refractive accommodative esotropia ,with history of surgery or extraocular muscle botulinum toxin injection were excluded.

After a detailed medical history had been obtained, preoperative assessments of vision, refractive error, ocular duction, vergence and stereopsis were obtained. The angle of deviation at 40 cm and 5 m was determined by alternate prism cover test .

In this prospective, non-randomized, controlled clinical study, patients were divided into two groups: a botulinum toxin group and a surgery group. Before formal treatment was initiated, all patient or their parents were given information about the patient’s condition and various treatment methods, including details related to the treatment process, cost, and possible benefits and risks. Patients voluntarily chose to undergo surgery or treatment with botulinum toxin. In principle, for patients ≥ 14 years of age, the degree of deviation in the botulinum toxin treatment group was not supposed to exceed 40 PD. However, if patients strongly desired to receive botulinum toxin treatment, we did not refuse. We did not have this restriction for patients younger than 14 years. This study was performed in accordance with the Declaration of Helsinki and was approved by the Ethics Committee of Wuhan Aier Eye Hospital (Hanyang), China (2017IRBKYA025). Written informed consent was obtained from all patients or their parents, in the case of patient age < 18 years.

### Treatment method

In the botulinum toxin group, patients received bilateral medial rectus injections of 4 IU for deviations < 30 PD, 5 IU for deviations of 30–40 D, 6 IU for deviations of 40–50 D, and 7 IU for deviations > 60 PD. Reinjection (4 IU) was offered whenever the patient had not achieved orthotropia ± 10PD, or diplopia had not disappeared by 5 months after the injection. If the patient was not cured after 3 doses, surgery was recommended.

For injections of botulinum toxin, patients under 14 years of age were given general anesthesia, and those over 14 years of age were given topical anesthesia. The patient was placed in a supine position. After anesthesia had been administered, the eyelids were opened, the medial rectus muscle was clamped, and the eye was rotated in abduction with tooth forceps. Botulinum toxin was injected 5–10 mm behind the insertion point of the muscle with a 27 G needle on an insulin syringe.

Surgeries were performed by the same doctor, with the patient under general anesthesia (patients ≤ 14 years of age) or local anesthesia (patients ≥ 14 years of age). The approach to surgery was determined by the maximum angle of deviation observed preoperatively. Bilateral medial rectus recession (MRC) was performed when the deviation was ≤ 60 PD); bilateral MRC + unilateral lateral rectus resection (LRR) was performed when the deviation was > 60PD. We increased the amount of resection on the basis of routine operation by 1–2 mm.If the patient complained of diplopia or slight residual esotropia immediately after surgery, adjustments were made within one week.

Patients were followed for at least six months. The criteria for successful treatment were the resolution of diplopia at distance and near and alignment within 10 PD or orthotropia. The standard of initial success was eye position within normal range within 6 months after the first extraocular muscle surgery or 1–3 bilateral botulinum injections. Subjects with residual deviations treated secondarily with sutures, botulinum toxin injection, or strabismus surgery were considered treatment failures, regardless of their final results. Diplopia and esotropia that reappeared after 6 months of AACE had been successfully treated were considered relapses. Those patients received additional injections of botulinum toxin or underwent reoperation. Deviation data at six months after primary treatment and at the last follow-up appointment (including further treatment for residual deviation and relapse) were recorded. The final number of successfully treated cases was defined as the initial number of successful cases minus the number of recurrences.

Patients who rejected our treatment were advised to reduce the amount of time devoted to near-work, to engage in binocular vision training at home, and to schedule regular follow-up visits.

SPSS version 23.0 (IBM SPSS Inc., Chicago, IL, USA) was used for statistical analysis. Differences in patient age, angle of deviation, and near work time were compared with the t-test. The chi-squared test was used for comparing treatment success rates and recurrence rates. When the number of cases was < 40, the theoretical number was < 5 cells and > 25% of the total number of squares, Fisher’s exact probability test was used.

## Results

During the study,76 patients were diagnosed with AACE at our hospital. Among them, 60 patients received treatment and completed follow-up. The average age was 22.3 ± 11.1 years. The study included 15 children (4–13 year, average 7.20 ± 3.83 year) and 45 adults and adolescents (14–57 year, average 24.7 ± 8.03 year). Forty-two patients were male, and 18 patients were female.

The vast majority of patients ≥ 14 years of age had a history of excessive near work, including time spent on smartphones, computers, writing, and reading. The average time devoted to near work was 11.4 ± 2.48 hours per day. Near work is not very common among children < 14 years of age, and average time devoted to near work among patients in this age group who were included in the study was only 2.93 ± 2.73 hours per day (t = 11.1, *P* < 0.001). Thirteen patients aged ≥ 14 years (28.80%) complained of significant mental stress from work or life.

Among all patients, 40 patients chose botulinum toxin injection, and 20 patients chose surgery. The other 16 patients chose to continue with observation.

Before treatment, sex ratio, age, and refractive status were similar in the botulinum toxin group and the surgical group. However, the degree of esotropia, vergence, and the time from symptom onset to presentation at the clinic were larger in the surgery group (See Table 1 for details).

**Table 1:** Preoperative data comparison between the surgery and botulinum toxin groups

	Botulinum toxin group (40)	Surgery group (20)	Chi-square or t-value	*P-*value
Age (years)	22.4 ± 11.2	18.0 ± 8.5	1.19	0.23
Sex (male:female)	28:12	14: 6	0.00	1.0
Right refraction (Diopter)	-1.40 ± 3.06	-1.72 ± 2.30	0.41	0.68
Left refraction (Diopter)	-1.38 ± 2.97	-1.90 ± 3.36	0.61	0.54
Setup time (Months)	9.60 ± 10.2	16.5 ± 13.5	-2.22	0.03
Deviation at distence (PD)	32.3 ± 15.4	44.0 ± 11.4	-2.98	0.004
Deviation at near (PD)	27.1 ± 13.8	39.6 ± 11.0	-3.48	0.001
Convergence (PD)	25.2 ± 6.78	44.2 ± 25.4	-4.44	0.00
Divergence (PD)	12.7 ± 5.02	22.2 ± 7.40	-5.85	0.00

*PD* Prism diopter

The botulinum toxin group included 31 patients ≥ 14 years of age (adult group) and 9 patients < 14 years of age (pediatric group). The average deviation and vergence before treatment in both groups are shown in Table 2. Deviation and vergence were larger in the pediatric group than in the adult group. However, the differences in deviation at distance and convergence were not significantly different between groups (*P* = 0.07 and *P* = 0.20).

**Table 2:** Deviation and vergence comparison between adults and children in the botulinum toxin group, before treatment

	Adult group (*n* = 31)	Children group (*n* = 9)	t value	*P* value
Deviation at distence (PD)	30.0 ± 13.7	40.5 ± 18.7	1.86	0.07
Deviation at near (PD)	24.3 ± 11.2	36.6 ± 18.2	2.48	0.01
Convergence (PD)	24.5 ± 6.87	27.7 ± 6.18	1.27	0.20
Divergence (PD)	11.8 ± 4.70	16.1 ± 4.85	2.39	0.02

*PD *Prism diopter

In the adult group, all 31patients had significant improvement in strabismus after the first injection of botulinum toxin. Among them, 21 cases (67.7%) achieved successful outcomes after at least 6 months of follow-up. Four patients still had esotropia > 10PD, and the diplopia did not disappear. We gave a second injection one month after the first injection. The other 6 patients were relieved within 1 month after the first injection, but relapsed at 1–5 months. After recurrences, we gave secondary injections. The average interval between the first and second injections and the first injection was 68.7 ± 32.9 days.After the second botulinum toxin injection, 8 of the 10 patients met the criteria for successful outcomes, but two female patients did not improve. We gave these two patients the third injection at about 3 months after the second injection, and they still had 20PD esotropia. We considered treatment to have failed in these 2 patients. Neither of the 2 patients who were not cured elected to pursue further treatment. The cumulative initial success rate was 93.5% in the adult group. See Table 3 for details.

**Table 3:** Therapeutic effects in the botulinum toxin group

Botulinum toxin group	Adult group (*n*=31)	Children group (*n*=9)	Total (*n*=40)	Chi square or t value	*P *value
Success cases for injection episode					
Frist injection (Number, %)	21 (67.7%)	8 (88.8%)	29 (72.5%)	/	0.41
Second injection (Number,%)	8 (25.8%)	1 (11.1%)	9 (22.5%)		
Third injection (Number,%)	0 (0%)	0 (0%)	0 (0%)		
Cumulative Initial success cases (Number,%)	29 (93.5%)	9 (100%)	38 (95%)	/	1.0
Relapse cases (Number,%)	9 (29.0%)	0 (0%)	9 (22.5%)	/	0.09
Final success cases (Number,%)	20 (64.5%)	9 (100%)	29 (72.5%)	/	0.04
Esophoria cases (Number,%)	18 (58.0%)	3(33.3%)	21 (52.5%)	/	0.26
Deviation at six month (PD)	6.12 ± 1.91	3.55 ± 3.60	5.53 ± 2.52	-2.86	0.007
Deviation at final follow-up (PD)	6.80 ± 1.88	4.22 ± 3.30	5.96 ±2.65	-3.01	0.005
Follow-up time (Months)	20.0 ± 8.64	15.1 ± 8.76	17.4 ± 5.64	-1.49	0.14

*PD* Prism diopter

In the pediatric group, 8 cases were treated successfully the first time, and 1 case was successful after the second treatment. The success rate was 100%. There was no significant difference in overall success rate between groups. Eighteen cases in the adult group (58.0%) had esophoria after treatment. Only three of the pediatric patients (30%) had esophoria (*P* = 0.26).

Nine patients (29.0%) in the adult group had recurrences after 6 months of follow-up. One case of relapse was observed in the pediatric group (*P* = 0.09). The final success rates in those two groups were 64.5% (20/31) and 100% (9/9).the difference between groups was statistically significant (Fisher’s exact test, *P* = 0.04). The two groups also differed significantly in the average angle of deviation at distance after six months of treatment and at the time of last follow-up (Table 3).

Of the 9 patients with recurrences, 8 received one botulinum toxin injection, and 1 patient received two injections. The recurrence time was more than 1 year after the initial resolution of symptoms. The average recurrence time was 15.6 ± 4.84 months. Moreover, these 9 patients with recurrences all had continued to engage in near-work over 10 hours per day after the initial resolution of symptoms (mean 15.4 ± 2.4 hours per day). Those 9 patients who experienced recurrence were given additional botulinum toxin injections. After injection, eye position returned to normal, and the patients’ diplopia disappeared. There was no recurrence during at least 3 months of follow-up.

Of the 20 patients in the surgery group, 6 (30%) were children < 14 years of age; 14 (60%) were teenagers or adults ≥ 14 years of age. Bilateral MRC was performed in 14 cases(70%), and bilateral MRC combined with unilateral LRR was performed in 6 cases (30%). Three patients (15%) underwent suture adjustment due to undercorrection ≤ 1 week after surgery, and 4 patients (20%) relapsed after six months of surgery. Among the 4 cases of recurrence, bilateral medial rectus injections of botulinum toxin were performed for 2 patients with deviation < 25 PD, and unilateral LRR surgery was performed in 2 patients with deviation > 30 PD. All patients recovered postoperatively, with no recurrence during at least 3 months of follow-up. When compared with the botulinum toxin group, the surgical group showed no significant differences in initial success rate, recurrence rate, final success rate, deviation at 6 months after treatment, or deviation at the last follow-up (Table 4).

**Table 4:** Comparison of the surgical and botulinum toxin groups in terms of therapeutic effects

Group	Botulinum toxin group (*n* = 40)	Surgery group (*n* = 20)	Total (*n* = 60)	Chi square or t value	*P* value
Initial success (Number,%)	38 (95%)	17 (85%)	39 (65%)	/	0.32
Relapse (Number,%)	9 (22.5%)	4 (20%)	13 (21.6%)	0.04	0.8
Final success cases (Number,%)	29 (72.5%)	13 (65%)	42 (70%)	0.35	0.55
Deviation at six month (PD)	5.55 ± 2.58	6.20 ± 2.28	5.53 ± 2.52	-0.95	0.34
Deviation at final follow-up (PD)	6.22 ± 2.48	6.50 ± 2.21	5.96 ± 2.65	-0.41	0.67
Follow-up time (Months)	18.9 ± 8.80	16.2 ± 13.4	17.4 ± 5.64	-0.94	0.35

*PD* Prism diopter

All 58 cured patients had normal stereopsis function, as determined by either random stereopsis or synoptophore examination. Seven patients (17.5%) in the botulinum toxin injection group experienced monocular ptosis of the upper eyelid about one week after injection, but all recovered spontaneously within 1–3 months. No other complications occurred. The patients who underwent extraocular muscle surgery had no other obvious complications except for slight scarring of the surgical incision.

## Discussion

In this study, 60 patients with AACE received our treatment. The study included significantly more male patients were than female patients (42:18).

In the past, most patients who received botulinum toxin therapy for AACE were less than 14 years old [9, 12]. Notably, Spierer defines the age range of adult acute esotropia as over 16 years old [20]. However, in our country, students over 14 years old have increased academic pressure, and the time devoted to reading and writing has increased significantly. So, we take 14 years of age as the cutoff between children and adults.

Excessive near work appeared to be an important risk factor for AACE inadu; among the population included in our study, the average time devoted to near work was 11.4 ± 2.48 hours per day. This value is similar to those reported previously [2, 4, 5]. Mental stress may also be involved in the occurrence of esotropia. However, for children < 14 years of age, the relationship between near work and AACE was not obvious. This type of AACE is more like late-onset concomitant esotropia in children [21]. We also found that convergence force was significantly greater than divergence force in these AACE patients. Sustained near work may enhance the force generated by the medical rectus muscles.

Patients with AACE are willing to choose botulinum toxin injection treatment because of its advantages: simplicity, convenience, minimal trauma, and low cost. In this study, 40 of 60 patients (66.6%) chose botulinum toxin injection treatment, including 31 adults and adolescents with age ≥ 14 years. .

Botulinum toxin was first used to treat strabismus in 1979 [18]. Currently it have been used to treat various types of strabismus. One of most important indications for botulinum toxin is the treatment of infantile esotropia [1, 2, 9, 11–13]. Of course, it is also used to treat pediatric acute-onset acquired esotropia [1, 2, 9, 12]. For example, Wan et al. [9] compared the effects of botulinum toxin injection vs. surgery in children with AACE. There were 16 patients in the botulinum toxin group and 33 patients in the surgery group. The results showed no significant difference in the success rate between groups at 6 months (81% vs. 61%, *P* = 0.20) or at 18 months (67% vs. 58%, *P* = 0.74). The median angle of deviation and median stereoacuity were similar between groups at 6 and 18 months.

Generally speaking, the younger a patient, and the smaller the angle of strabismus, the better the treatment effect [11, 12, 16, 17, 19]. The effects of botulinum toxin in infants and children with squint are quite good, but the effects of botulinum toxin when used to treat constant squint in adults are uncertain. Recently, we found some authors reported the use of botulinum toxin to treat adult AACE [1, 2]. Wang [1] described 6 AACE patients, aged 3–34 years, who were treated with botulinum toxin. The study included 3 patients aged ≥ 14 years. The overall success rate in the study was 83.3 (5/6). Another recent study of 13 patients with AACE (age 3–24 years, average age 12.61 ± 6.74 years) treated with botulinum toxin injection included adult patients [2]. Although the authors do not clearly indicate those adult patients treatment results, we speculate that botulinum toxin was effective for them. So far, we have not found a study focused on botulinum toxin in the treatment of adult AACE.

In our study, 40 patients with AACE chose botulinum toxin injection therapy, including 31 patients ≥ 14 years of age. After three doses of treatment, only 2 adult patients did not meet the criteria for cure. The treatment success rate reached 95%. Certainly, the treatment effect was not as great as observed in patients younger than 14 years of age. In the adult group, the residual angle of deviation after treatment was higher than in the pediatric group; 18 cases (58.0%) had esophoria after treatment, and 9 patients (29.0%) relapsed after 6 months of treatment. In the pediatric group, all 9 patients reached orthotropia after two dose of botulinum toxin injection, and no patients had relapse.The final treatment success rate was significantly different between the two groups( 64.5% vs. 100%) .We speculate that, with an extended follow-up period, the chance of recurrence in the adult group may continue to increase. We believe that relapse in these patients is related to residual implicit deviation on the one hand, and continued excessive use of mobile phones on the other.

In general, the efficacy of botulinum toxin in the adult group was satisfactory. In addition to the mechanism of action of botulinum toxin that have reported by other studies [22–24], we speculate that it is also related to the characteristics of AACE. First, most adult patients with AACE had a relatively small esotropia angle. Second, the time elapsed since symptom onset was brief. AACE is an acute strabismus. The time from onset to treatment is generally within 2 years, usually about a few months. Therefore, after treatment, the extraocular muscles have a greater chance of remodeling and recovery. Other types of adult strabismus often start in childhood. Third, these patients have the potential for stereopsis. After treatment, stereopsis is re-established and plays a role in controlling eye position, so that the treatment effect can be sustained. In this study, all cured patients had relatively normal stereopsis after treatment, as determined by synoptophore or random-dot stereopsis pictures.

Only 20 patients in this study chose extraocular muscle surgery. Those patients had relatively large angles of esotropia (mean 44.0 ± 11.4 PD at distance and 39.6 ± 11.0 PD at near).

In the early years of AACE surgery, the undercorrection rate was very high, reaching 75%. Many of these patients required additional strabismus surgery. The reasons may be related to the prism-eating phenomenon caused by mechanisms that compensate for the deviation. Application of the prism adaptation test (PAT) significantly improved the success rate of surgery in AACE patients [6–8]. However, the PAT itself requires a long time, especially for patients with high strabismus angles. It is difficult for these patients to wear prisms for several days. Some authors, including us, prefer to reduce the incidence of undercorrection and avoid the time and expense associated with PAT by increasing the amount of the correction during surgery [2, 3, 10]. We increased the amount of surgery by 1–2 mm beyond what is typical for esotropia surgery.The results showed that the treatment effect was similar to that achieved with botulinum toxin injection. Three of the 20 patients (15%) underwent suture adjustment due to the undercorrection of esotropia, and 4 patients(20%) relapsed after half a year. Only 13 patients (65%) had satisfactory results after one operation. Therefore, it is still a challenge for strabismus correction doctors to determine the target surgical volume more conveniently and accurately for AACE patients.

In this study, 7 patients (17.5%) in the botulinum toxin injection group experienced monocular ptosis of the upper eyelid about one week after injection, and all recovered spontaneously within 1–3 months. No other obvious complications occurred. Therefore, surgery as well as botulinum toxin injection are safe for AACE patients.

During the same period, 16 patients who did not choose surgery or botulinum toxin treatment were followed through in-person visits to the hospital or via telephone. None of the symptoms of these patients improved. A study has reported that the esotropia of some patients may be improved or even resolved by reducing the time spent using mobile phones [5]. This phenomenon did not appear in our patients. One of the important reasons may be that people are becoming more and more reliant on electronic products, especially smartphones, and it is difficult to effectively reduce the time spent on near-work.

This study has some limitations. First of all, for ethical reasons, we did not randomly group patients, resulting in a relatively small number of surgical patients. There was a significant difference in the angle of deviation between groups before treatment, which made the comparison of treatment effect between groups difficult. In addition, the follow-up period was not sufficiently long, and the long-term stability of these treatment effects needs further observation.

## Conclusion

In this prospective, nonrandomized, controlled clinical study, excessive near work was identified as an important risk factor for AACE in patients ≥ 14 years of age. The injection of botulinum toxin has a good effect on AACE in adults with minimal and moderate squinting and in children. The outcomes achieved with injected botulinum toxin are similar to those achieved with surgery.

## Data Availability

The data are available from the corresponding author upon reasonable request.

## Abbreviations

AACE: Acute acquired concomitant esotropia
PD: Prism diopters
SE: Spherical equivalents
BCVA: Best-corrected visual acuity
OD: Right eye
OS: Left eye
MRC: Medial rectus recession
LRR: Lateral rectus resection
